# Epigenetic Landscapes in Ulcerative Colitis: From Mechanistic Insights to Clinical Translation

**DOI:** 10.1021/acsomega.5c07523

**Published:** 2025-10-27

**Authors:** Linhui Li, Xiaofen Luo, Yang Tang, Fei Tang, Feng Zhou, Chen Sun, Dongqin Huang, Zewei Li, Yang Tan, Ling Li

**Affiliations:** † School of Pharmacy, 118393 Hunan University of Chinese Medicine, Changsha 410208, China; ‡ Key Laboratory of Modern Research of TCM, Education Department of Hunan Province, Changsha 410208, China; § National Key Laboratory Cultivation Base of Chinese Medicinal Powder & Innovative Medicinal Jointly Established by Province and Ministry, Hunan University of Chinese Medicine, Changsha 410208, China; ∥ Shaanxi Collaborative Innovation Center of Chinese Medicinal Resources Industrialization, State Key Laboratory of Research and Development of Characteristic Qin Medicine Resources (Cultivation), Shaanxi University of Chinese Medicine, Xianyang 712046, China

## Abstract

Ulcerative colitis (UC) is distinguished by nonspecific inflammation and ulceration of the rectal and colonic mucosa. The conventional therapeutic modalities for UC exhibit restricted efficacy, underscoring the importance of advancing novel mechanisms and therapeutic interventions. The field of epigenetics, encompassing DNA methylation, histone modification, noncoding RNAs (ncRNAs), and RNA modification, is progressively illuminating the association with UC. Moreover, the current focus of intense scrutiny in drug development is the utilization of epigenetics as a potential target for anti-UC therapy. Significantly, the aberrant epigenetic modifications exert influence over various behaviors exhibited by intestinal epithelial cells and inflammatory cells. In this review, we have examined and synthesized current clinical and experimental research to comprehensively analyze the expression patterns and elucidate the underlying mechanisms of epigenetic alterations in the advancement of UC, with a particular focus on DNA methylation, histone modification, ncRNAs (miRNA, lncRNA, and circRNA), and RNA modification. Additionally, we discussed the obstacles and the potential future directions related to epigenetic modifications in UC. Deciphering the intricate epigenetic modifications in UC offers potential for yielding novel understandings of the disease’s etiology and pathogenesis and the identification of diagnostic and therapeutic targets, thereby establishing a basis for achieving optimal outcomes in UC.

## Graphical abstract

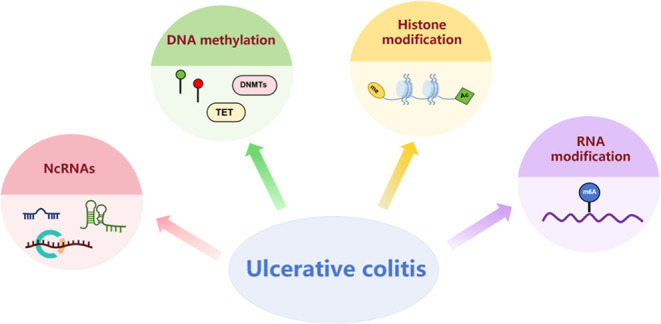

Ulcerative colitis (UC) is a persistent, immune-mediated inflammatory disorder of the colon, characterized by a multifaceted etiology.^1^ The primary clinical symptoms of UC encompass diarrhea, hematochezia, and abdominal discomfort. The protracted nature of UC, coupled with its numerous complications, significantly impairs the overall health and life quality of the affected individuals.^2^ In the past decade, the incidence of UC has been increasing dramatically. At present, although there are some drugs currently approved for use, such as steroids, antitumor necrosis factor (TNF) biologics, vedolizumab, and small molecule Janus kinase (JAK) inhibitors, they are not fully efficacious in all patients.^3^ Exploration of epigenetics of the UC process will help personalize medicine and choose the right drug for individual patients.

Epigenetics is a scientific discipline that investigates alterations in gene expression, which do not involve changes to the underlying nucleotide sequence. Epigenetics is the scientific discipline that investigates heritable modifications in gene expression regulation that do not involve changes in the underlying nucleotide sequence. This field primarily encompasses regulatory mechanisms, such as ncRNAs, DNA methylation, histone modification, and RNA modification-based processes. Researchers are increasingly recognizing that epigenetic changes are closely related to specific complex diseases, such as metabolic diseases, Alzheimer’s disease, and cancer.^4–6^ UC is caused by complicating pathogenesis, involving genetic, immune, intestinal flora, and many other factors. Epigenetics is an important interpretive link connecting the complex interactions between genetics and external risk factors that lead to the development of UC. It is believed that the dysregulation of epigenetics will lead to the development of UC.^7^

In this review, we provide a comprehensive overview and synthesis of current clinical and experimental research examining the regulators of epigenetic modification and their associated mechanisms in the initiation and progression of UC. Our primary emphasis is on DNA methylation, noncoding RNAs, histone modification, and RNA modification. High-throughput detection techniques, biological enrichment discovery, and CRISPR/Cas are strategically utilized to analyze and explore epigenetic modifications in UC, followed by corroboration through experimental validation. Understanding the complex epigenetic changes in UC may provide new understanding of the molecular processes involved in UC development and establish a basis for accurate diagnosis and treatment of the UC. Moreover, the current research demonstrates that epigenetics may serve as a potential therapeutic target for UC and be used as a diagnostic tool to differentiate between UC and Crohn’s disease, as well as to predict the predisposing factors for the progression of UC to colon cancer.

The mucosa of the colon protects the underlying tissues and is exposed to millions of antigens from the food, environment, and microbiome.^1^ Intestinal epithelial cells (IECs) and tight junctions (TJs) form the first line of defense of the colonic mucosa. In UC, this defend line was disturbed, which leads to the increased permeability of the mucosa to luminal pathogens. As the imbalance between IECs proliferation and death is the inevitable event of UC, how epigenetics contributes to UC formation by modulating this balance has become a hot topic. Due to the increased permeability of the mucosa, those antigens are able to invade the submucosa and then stimulate immune cells. Subsequently, immune cells, including macrophages, dendritic cells, and T cells, drive the excessive immune response, which stimulate an inflammatory cascade and then aggravate UC.^8^ In this condition, the aberrant immune response is governed by an array of epigenetic mechanisms that participate in the pathogenesis of UC via a sophisticated, dynamic regulatory network. The epigenetic modification pathways demonstrate potential for the therapeutic management of UC (Figure 1). Additionally, a comprehensive comprehension of the involvement of epigenetics in UC may aid in the timely identification of the condition, differentiation between UC and Crohn’s disease, and anticipation of cancer progression.^9^

**Figure 1:**
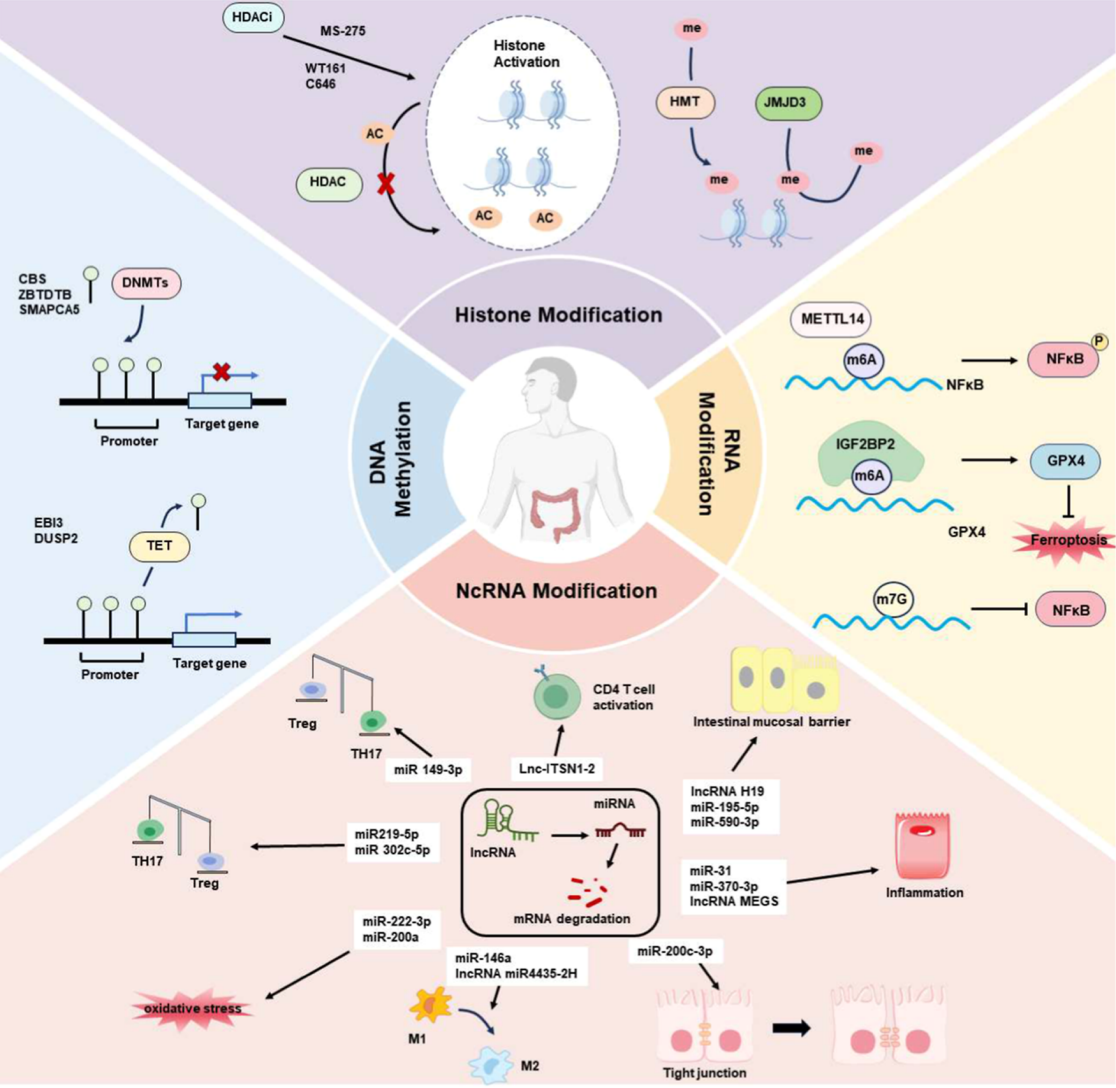
Effects of epigenetic modifications on UC.

## Why Is UC Related to Epigenetics?

The human gastrointestinal system harbors the greatest abundance and variety of microbiota. The enteric microbiota typically coexist in a symbiotic relationship with the host, performing crucial functions in digestion, the development of the immune system, and the preservation of epithelial barrier integrity. Concurrently, the microbiota functions as an environmental monitor, capable of promptly reacting to external stimuli such as alterations in diet or the environment. A clinical investigation examined the microbiota, host transcriptomics, epigenomics, and genetics in inflamed and noninflamed colonic mucosa, revealing that epithelial DNA methylation enhances disease categorization and exhibits a correlation with inflammation and the composition of the microbiota.^10^ When UC occurs, the composition of the gut microbiota is altered and can also affect the host by secreting extracellular vesicle (EV).

Indeed, the microbiota also has the capacity to produce numerous biological compounds, some of which function as epigenetic substrates, cofactors, or regulators of epigenetic enzyme activity.^11^ The interactions between gut microbiota and its metabolites are intricately associated with DNA methylation, histone modification, and the regulation of noncoding RNAs (ncRNAs).^12^ Particularly, butyrate, an essential short-chain fatty acid within the intestinal flora, has been shown to play a crucial role in promoting iTreg differentiation through the facilitation of histone acetylation, thus contributing significantly to intestinal homeostasis.^13^ A functional peptide of p40, derived from *Lactobacillus rhamnosus*, has been shown to induce upregulation of Setd1b, a gene encoding a methyltransferase. This action promotes both mono- and trimethylation of histone 3 at lysine 4 (H3K4me1/3). Furthermore, it enhances the expression of the Tgfb gene in IECs, contributing to a sustained preventative effect against colitis.^14^

Immune cells represent an additional source susceptible to epigenetic modifications during the progression of UC, as they adapt their functions through alterations in metabolic pathways in response to shifts in the tissue microenvironment to uphold tissue homeostasis.^15^ Substantial heterogeneity is observed among various immune cell types in individuals with UC. These modifications are epigenetically primed for macrophage differentiation.^16^ In Treg cells from UC patients, there is a sustained activation of the Wnt-β-catenin pathway, which leads to the induction of newly accessible chromatin sites within proinflammatory genes, resulting in an upregulation of their expression, thus promoting the disease-associated Treg phenotype^17^ (Figure 2).

**Figure 2:**
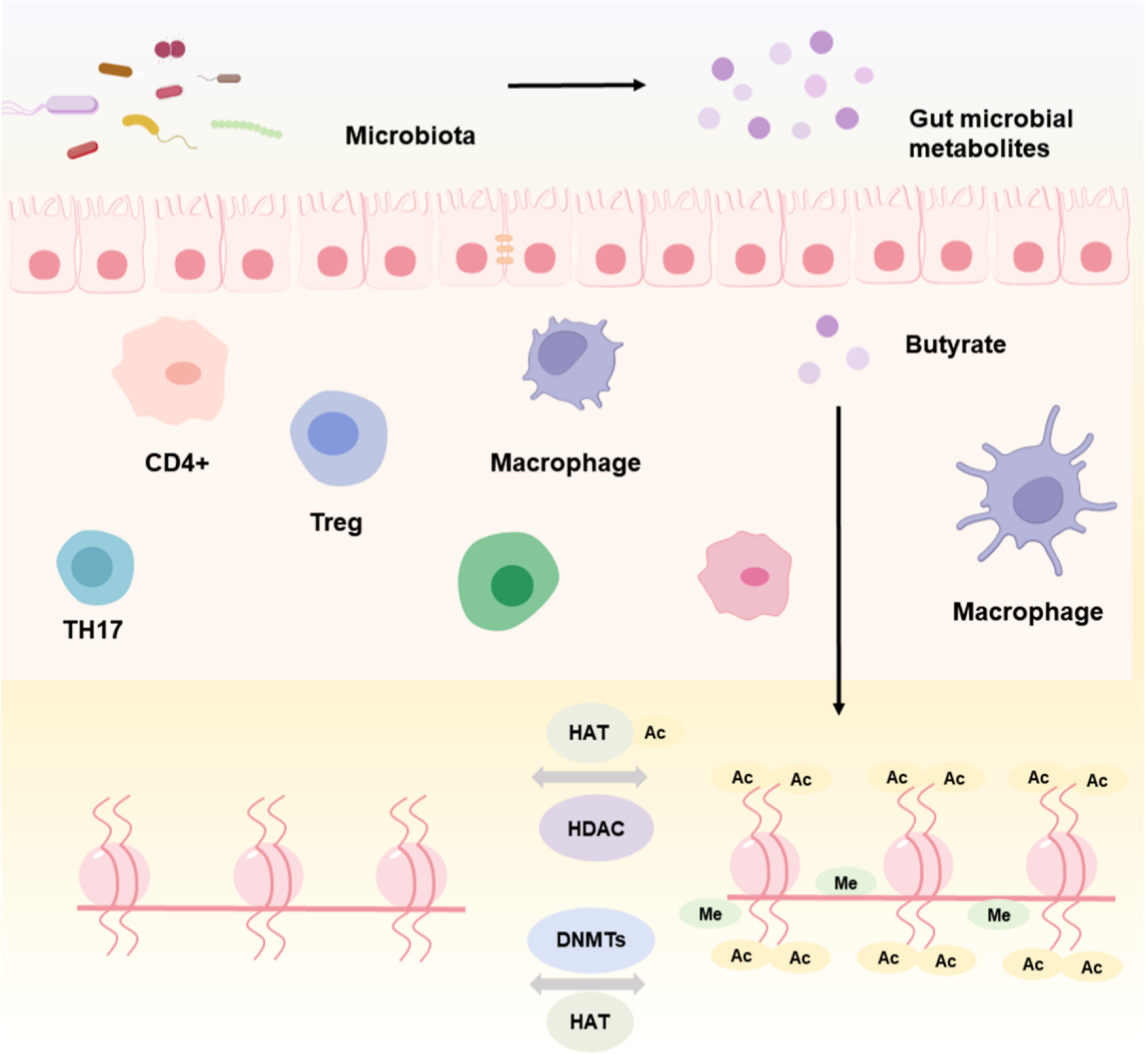
Gut microbiota exerts an influence on the development of ulcerative colitis via mechanisms of epigenetic inheritance.

## ncRNAs in UC

### ncRNAs as the Diagnostic and Prognostic Biomarkers of UC

ncRNAs are a category of RNAs that are not translated into proteins but function to regulate gene expressions at both the transcriptional and post-transcriptional levels, including microRNAs (miRNAs), long noncoding RNAs (lncRNAs), and circular RNAs (circRNAs).

miRNAs are endogenous, single-stranded ncRNAs consisting of 17–25 nucleotides.^18^ They specifically target the 3′ untranslated region of a given gene and either inhibit or degrade the target gene, depending on the degree of complementary bases. miRNAs are considered potential targets for the therapeutic treatment of UC.^19^ However, the difficulty lies in the fact that a single miRNA has the ability to control numerous proteins, making it a challenge to minimize the impact on nontarget proteins.^20^ Researchers have identified potential biomarkers that differentiate between UC and Crohn’s disease (CD) in both biopsies and blood samples miRNAs obtained from patients diagnosed with these two diseases.^21,22^ The levels of miRNAs were also found to be associated with patient clinical characteristics, including positive and negative correlation.^23^ Specific miRNAs, which were in rectal mucosal samples from UC patients with dysplasia or CRC, have potential to be biomarkers to identify patients with UC who are prone to developing colorectal cancer, and preventing morbidity and mortality from this chronic disease.^24^ Moreover, a strong correlation exists between the reduction in miR-206 levels and positive changes in the tissue structure in individuals with UC receiving long-term treatment with 5-ASA, indicating its potential as a valuable biomarker for predicting response to mesalamine therapy.^25^

The disturbance expression of lncRNAs, linked with the disorders of gut microbes and mucosal homeostasis, shows a strong association with the severity and outcome of UC.^26^ lnc-ITSN1-2 gene is demonstrated as a strong predictive value for the risk of inflammatory bowel disease (IBD), particularly in cases of active disease, and exhibited positive correlations with disease activity and inflammation markers in IBD patients. Further mechanistic studies revealed that lnc-ITSN1-2 functions as a competing endogenous RNA (ceRNA) by directly binding to miR-125a to upregulate IL-23R expression. Through this regulatory pathway, lnc-ITSN1-2 modulates CD4 T cell functions in IBD.^27^

circRNAs are generated by reverse splicing of exons or introns from mRNA precursors and exhibit variable expression levels across different tissues. Among these, circSOD2 is abundantly expressed in the colonic mucosa of patients with UC and plays a crucial role in epithelial barrier repair via the miR-378g/Snail1 axis.^28^ Furthermore, the expression levels of circRNA_103516 in peripheral blood mononuclear cells (PBMCs) hold potential as a diagnostic biomarker for IBD.^29^ Similarly, circCCND1 mitigates the progression of UC by acting as a sponge for miR-142-5p, highlighting its potential both as a therapeutic target and a novel biomarker in UC.^30^ These research studies provide evidence of ncRNAs as biomarkers for disease and effectiveness of drug treatment (Table 1).

**Table 1:** Mechanisms of ncRNAs in UC (Study Type: Clinical)^a^

years	numbers of clinical samples	sample sources	ncRNA expression (expression trend in UC)	clinical value	ref.
2017	CD (*n* = 12)	plasma	upregulated: miR-598 and miR-642	distinguish UC and Crohn’s colitis	^21^
	UC (*n* = 21)				
2021	new pUC (*n* = 15)	rectum biopsies	upregulated: miR-21 and miR-126	correlated miRNA expression with histologically and endoscopically assessed severity of disease	^23^
	known pUC (*n* = 15)				
	new aUC (*n* = 15)		downregulated: miR-31 (all patients), miR-142, and miR-155(pediatric patients only)		
	known aUC (*n* = 15)				
2017	control (*n* = 90)	tissue specimens collected from patients	upregulated: miR-1, miR-9, miR-124, and miR-137	as diagnostic biomarkers for identifying patients at higher risk of UC-CRC and as novel biomarkers for identifying patients who are at a higher risk of UC-associated dysplasia or cancer	^24^
	UC (*n* = 61)				
2019	control (*n* = 90)	colonic biopsy specimens	downregulated: miR-206	significantly elevated in active UC biopsy tissue, and treatment with 5-ASA downregulates its expression in colonic mucosa	^25^
	UC (*n* = 61)				
2023	control (*n* = 20)	mucosa	downregulated: GATA6-AS1, CDKN2B-AS1, HNF1A-AS1 et al.	associated with gut microbes and mucosal homeostasis, and potential targets	^26^
	UC (*n* = 206)				
	SEEM (*n* = 42)				
2020	control (*n* = 30)	mucosa samples	upregulated: lnc-ITSN1-2	expressions were increased in IBD patients compared to HCs, and presented with good predictive values for IBD risk	^27^
	A-UC (*n* = 30)				
	R-UC (*n* = 30)				
	A-CD (*n* = 30)				
	R-CD (*n* = 30)				
2024	control (*n* = 20)	colonic mucosal	upregulated: circSOD2	the expression in patients with active ulcerative colitis was significantly higher than that in patients with inactive UC	^28^
	active UC (*n* = 12)				
	nonactive UC (*n* = 8)				
2019	control (*n* = 80)	peripheral blood mononuclear cells	upregulated: circRNA_103516	the level of circRNA_103516 in PBMC can be regarded as an ideal candidate biomarker for diagnosing IBD	^29^
	UC (*n* = 90)				
	CD (*n* = 90)				
	PC (*n* = 35)				
2023	control (*n* = 20)	colonic mucosa	downregulated: circRNA CCND1	the circRNA CCND1, through miR-142-5p, alleviated the progression of UC, indicating that the circRNA CCND1 can serve as a novel biomarker for UC	^30^
	UC (*n* = 20)				
2015	UC (*n* = 17)	tissues and serum of patients	upregulated: miR-26b	potential biomarker for inflammation-associated processes in the gastrointestinal system, and discriminated between UC-associated colorectal carcinoma (UCC) and the sporadic cancer type	^31^
	UCC (*n* = 38)				
2021	control (*n* = 10)	serum and colon tissue samples from patients	upregulated: miR-506	clearly differentiates patients with PSC + UC from patients with UC alone, and different phenotypic presentation of colitis may be related to miR-506 expression	^32^
	UC (*n* = 10)				
	PSC (*n* = 10)				
	PSC + UC (*n* = 10)				
2019	control (*n* = 33)	colonic biopsies and blood samples	upregulated: miR-24	reduce miR-24 levels in actively inflamed UC patients which could strengthen the intestinal barrier	^33^
	UC (*n* = 65)				
	IBS (*n* = 18)				
	CD (*n* = 28)				
2022	control (*n* = 50)	biopsies	upregulated: miR-31, miR-106a, and miR-135b	distinguish between colitis-related dysplasia and colorectal cancer and facilitate the screening and early treatment of precancerous lesions	^34^
	UC-NM (*n* = 20)				
	UC-Dys (*n* = 20)				
	UC-CRC (*n* = 12)				
2020	control (*n* = 23)	stool samples	upregulated: miR-223 and miR-1246	the first comprehensive screen of faecal miRNAs performed in IBD, and active UC patients displayed significantly higher levels than controls	^35^
	UC (*n* = 35)				
	CD (*n* = 44)				
	CDI (*n* = 8)				
2023	control (*n* = 24)	serum and colon tissues	upregulated: miR-129-2-3p	serum exosomes miR-129-2-3p may serve as a sensitive and specific biomarker for the diagnosis of UC and Fn-infected UC	^36^
	UC (*n* = 72)				
2017	control (*n* = 12)	tissue specimens from patients	downregulated: miR-193a-3p	downregulate in UC neoplasia, and a potential target for future chemopreventive approaches in this high-risk population	^37^
	UC (*n* = 9)				
	UC with neoplasia (*n* = 11)				
2014	CC (*n* = 12)	biopsies	upregulated: miR-146a and miR-21	significantly enhanced in UC patients compared to UC remission	^38^
	UC (*n* = 16)				
	LC (*n* = 13)				
2015	control (*n* = 107)	colon specimens	upregulated: miR-214	miR-214 is an epithelial gene that is deregulated in UC patients with active disease compared to those in remission, and it regulates NF-κB activity	^39^
	UC (*n* = 120)				
2018	control (*n* = 40)	mucosa samples	downregulated: miR-449a	expressed in a decreased pattern during the neoplastic transformation of CAC	^40^
	UC (*n* = 63)				
	CD (*n* = 49)				
	CAC (*n* = 41)				

a Annotation: new pUC: de novo diagnosed pediatric patients, known pUC: previously diagnosed pediatric patients, new aUC: de novo diagnosed adult patients, known aUC: previously diagnosed adult patients, SEEM: people with SEEM type celiac disease, A-CD: active CD, R-CD: remission CD, A-UC: active UC, S-UC: remission UC, PC: patient controls, UCC: UC-associated colorectal carcinoma, PSC: primary sclerosing cholangitis, IBS: diagnostic Rome III criteria for IBS, UC-NM: UC normal mucosa biopsies, UC-Dys: UC-associated dysplastic lesions, UC-CRC: UC-associated colorectal cancers, CDI: patients with *Clostridium difficile* infection, CC: noninflamed controls and patients with active or in-remission collagenous colitis, LC: lymphocytic colitis.

### Mechanisms of ncRNAs in UC

#### Anti-UC ncRNAs

miR-31, which is reported to be increased in colon tissues from UC patients, acts as a defender to protect against UC by preventing the expression of inflammatory cytokine receptors Il7R and Il17RA and GP130 signaling proteins. miR-31 also promotes epithelial regeneration following injury through regulating WNT and Hippo pathways.^41^ miR-370-3p in UC mice has been shown to attenuate epithelial–mesenchymal transition (EMT) by inhibiting inflammatory pathways and modulating β-catenin signaling, consequently leading to a decreased expression of tumor-associated proteins.^42^ Additionally, the upregulation of miR-195-5p has been observed to enhance the expression of claudin-2, thereby reducing colonic permeability and maintaining the integrity of the mucosal barrier, which confers a protective effect in experimental acute colitis.^43^ Another M2 macrophage-derived extracellular vesicles (M2-EVs)-derived miRNA, miR-590-3p, has been discovered to repair the injured IECs and then improve DSS-induced colitis.^44^ miR-24-3p is revealed to suppress IEC apoptosis targeting of BIM, a proapoptotic member of the Bcl-2 family, and plays a protecting role to damaged mucosa in UC.^45^ miR-574-5p in IECs is downregulated by *Fusobacterium nucleatum*-derived extracellular vesicles, which is followed by the aggravating experimental UC in mice through CARD3-dependent autophagy activation.^46^

Immune cells are also the target cells of miRNAs against UC.miR-146a, from the colon explant and DC-derived exosomes, is targeted binding to the key proinflammatory regulators such as TNF receptor associated factor 6, interleukin 1 receptor-associated kinase 1, and NLR family pyrin domain containing 3 (NLRP3), then promotes the transition of intestinal macrophages to the anti-inflammatory phenotype, thus reduces intestinal inflammation during colitis.^47^ The expression of miR-219-5p is downregulated in colorectal cancer, and the upregulation of miR-219-5p is associated with a decreased proportion of T-helper type 17 (TH17) cells, thus reducing UC inflammatory damage.^48^

Of course, other kinds of ncRNAs are also involved in the above process. The level of lncRNA H19, upregulated by IL22, is required for promoting the proliferation of IECs and epithelial regeneration by inhibiting p53 protein, miR-34a, and let-7.^49^ In a mouse UC model, circRNA-circPan3 is reported to promote the self-renewal ability of intestinal stem cells (ISCs) through the IL-13–IL-13R signaling pathway, mediated by type 2 innate lymphoid cells in the crypt microenvironment.^50^

#### ncRNAs Involved in Promoting UC

However, the increased expression of some miRNAs plays the “bad roles” to exacerbate UC damage by disrupting the intestinal mucosal barrier. The upexpressed miR200C-3p is reported to be involved in the process when IL1B increases the permeability of intestinal TJs.^51^ miR-149-3p, from the exosomes-derived *Bacteroides fragilis*-treated cells, is revealed to facilitate the differentiation of TH17 cells, finally induced UC-associated colorectal carcinogenesis.^52^ Another miR-302c-5p is also revealed to suppress the differentiation of TH17 cells, leading to a downward trend of producing proinflammatory cytokines, thus ameliorating the progression of UC. The mechanism of this action is believed to be related to the interaction between miR-302c-5p and STAT3.^53^ A study revealed the inhibiting of miR-222-3p in IECs mitigates oxidative damage by targeting BRG1, which subsequently activates the Nrf2/HO-1 signaling pathway, thereby reducing colonic inflammation.^54^ However, miR-200a, which is also downregulated in UC tissues, can activate the Nrf2-regulated antioxidant pathway targeting Keap1 to protect against DSS-induced UC damage.^55^ Moreover, lncRNA NAIL is revealed to inhibit the activity of Wip1 phosphatase, consequently alleviating its suppression of p65, thereby promoting the differentiation of precursor cells in the bone marrow into immature myeloid cells, recruiting macrophages to the inflammatory area, and promoting expression of inflammatory genes in colitis.^56^ On the contrary, the suppression of lncRNA miR4435-2HG inhibits macrophage M1 polarization and facilitates M2 polarization, consequently mitigating UC through JAK1/STAT1 signaling.^57^

#### The Interactions between miRNA and lncRNA in UC

Part of lncRNAs have the abilities to sequester miRNAs, forming complexes that hinder the functionality of the miRNA and consequently influence the expression of target genes. This process aligns with the ceRNA theory, which suggests that lncRNAs could competitively bind specific miRNAs through their miRNA response elements, ultimately impacting the regulation of genes associated with the etiology of certain conditions such as UC.^58^ lncRNA H19 has been shown to act as a sponge for miR-675-5p, leading to the downregulation of key biomarkers associated with intestinal epithelial barrier function, including the expression of vitamin D receptor and zonula occludens-1. This regulatory mechanism ultimately contributes to the onset of UC.^59^ lncRNA NEAT1 is markedly increased in IECs upregulated in IECs from UC patients. As the sponge of microRNA-410-3p, NEAT1 downregulates the expression of microRNA-410-3p in IECs. Moreover, further experiments demonstrated that the reintroduction of miR-410-3p in NEAT1-overexpressing IECs effectively reversed the NEAT1-induced cell death in response to LPS treatment.^60^ lnc-RNA MEG3 derived from M2-EVs is reported to competitively bind to miR-20b-5p within colon epithelial cells, leading to the decreased inflammatory reactions in UC.^61^

It should be noted that the role of ncRNAs in UC may vary depending on the disease stage, cell type, and tissue microenvironment. Furthermore, multiple research studies have shown that the function of lncRNA ANRIL is highly context-dependent. This indicates that ANRIL does not perform a single function but may regulate different downstream targets and signaling pathways through the adsorption of various miRNAs, such as miR-323b-5p and miR-195-5p, in different physiological and pathological contexts, ultimately leading to distinct biological effects.

The roles of some ncRNAs in the development of UC have been shown to have both positive and negative effects. lncRNA ANRIL has been proven to be involved in the regulation of the inflammatory pathogenesis of UC. In the serum sample from UC patients, the expression level of LncRNA ANRIL is negatively correlated to inflammatory cytokines. It acts as a molecular sponge, specifically binding and inhibiting the activity of miR-16 and miR-195, thereby relieving the inhibitory effect of these two miRNAs on antiapoptotic target genes, further enhancing the survival ability of IECs.^62^ However, lncRNA ANRIL is also proved to be highly expressed in the colonic mucosal tissues of UC patients, and negatively correlated with the anti-inflammatory miR-323b-5p, thereby activating the classical inflammatory signaling pathway of TLR4/MyD88/NF-κB, inducing the massive release of proinflammatory cytokines, and exacerbating the inflammatory pathological process of colitis.^63^ Another study has shown that the downregulation of lncRNA ANRIL expression in the UC model leads to a weakened adsorption ability for miR-191-5p, unable to effectively inhibit the expression of the target gene SATB1 of this miRNA, thereby promoting the excessive production of proinflammatory factors such as IL-6 and TNF-α, driving and worsening the inflammatory response of UC.^64^

The role of ncRNAs in the progression of UC has been revealed to some extent (Table 2). Among those, researchers are still mainly focused on the intervention mechanisms of miRNAs on the mucosal barrier and immune cells in the colon tissues, and the reports on the involvement of lncRNAs and circRNAs in UC are still limited.

**Table 2:** Mechanisms of ncRNAs in UC

years	sample source	ncRNAs (expression trend in UC)	pathways	biological effects	ref.
2019	human and DSS/TNBS treating mice	upregulated: miR-31	WNT and Hippo pathways	prevention of expression of inflammatory cytokine receptors Il7R and Il17RA and GP130 signaling proteins	^41^
2020	DSS/AOM treating mice	downregulated: miR-370-3p	β-catenin signaling	inhibition of inflammatory processes reduced expression of tumor-associated proteins	^42^
2022	DSS treating mice	downregulated: miR-195-5p		increases claudin-2 expression, thereby reducing colonic permeability and maintaining mucosal barrier integrity	^43^
2021	DSS treating mice	downregulated: miR-590-3p	LATS1/YAP/β-catenin signaling axis	discovered to repair the injured IECs	^44^
2021	DSS treating mice	downregulated: miR-24-3p	BIM	exacerbation of experimental UC in mice by activation of autophagy in a CARD3-dependent manner	^45^
2023	DSS treating mice	downregulated: miR-574-5p	miR-574-5p/CARD3 axis	exacerbation of experimental UC in mice by activation of autophagy in a CARD3-dependent manner	^46^
2022	human and DSS treating mice	upregulated: miR-146a		reduce intestinal inflammation in colitis by promoting the transition of intestinal macrophages to an anti-inflammatory phenotype	^47^
2020	DSS treating mice	downregulated: miR-219-5p		contribute to a reduction in the proportion of TH17 cells, thereby reducing inflammatory damage in UC	^48^
2018	human and DSS/LPS treating mice	upregulated: lncRNA H19	P53	promotion of IEC proliferation and epithelial regeneration	^49^
2020	DSS treating mice	upregulated: miR200C-3p		increases the permeability of the intestinal tight junction	^51^
2021	human and DSS treating mice	downregulated: miR-149-3p		facilitation of TH17 cell differentiation, ultimately inducing UC-associated colorectal carcinogenesis	^52^
2022	DSS treating mice	upregulated: miR-302c-5p	miR-302c-5p/STAT3 axis	suppress differentiation of TH17 cells, resulting in reduced synthesis of proinflammatory cytokines	^53^
2023	DSS/AOM treating mice	upregulated: miR-222-3p	Nrf2/HO-1 signaling	attenuate oxidative damage	^54^
2023	DSS treating mice	downregulated: miR-200a	Keap1/Nrf2 signaling	regulate antioxidant pathway	^55^
2023	DSS treating mice	upregulated: lncRNA miR4435-2HG	JAK1/STAT1 signaling	inhibition of macrophage M1 polarization while promoting M2 polarization	^57^
2016	DSS treating mice	upregulated: lncRNA H19		downregulated the biomarkers of intestinal epithelial barrier function, such as VDR and ZO-1	^58^

### Medications Used to Treat UC Affect ncRNAs

Limonin, a triterpene extracted from *Citrus aurantium*, has demonstrated anti-inflammatory and apoptotic properties, during UC, by suppressing STAT3/miR-214 signaling.^65^ Cinnamaldehyde exhibited the potential efficacy in treating UC by suppressing the activation of NLRP3 inflammasome and the expression of miR-21 and miR-155 in both colonic tissues and macrophages.^66^ *Clostridium butyricum*-derived extracellular vesicles (CbEVs) are revealed to restore the expression of miR-199a-3p, inhibiting the signaling of proinflammatory MAPK and NF-κB signaling, thus alleviating the imbalance of intestinal flora in colitis mice, following the regulation of the metabolism of tryptamine and the maintenance of the integrity of the intestinal mucosal barrier.^67^

Novel formulations based on ncRNAs have been widely used to combat UC damage. The above-mentioned miR-31 has been incorporated into OKGM peptosome microspheres to target the colonic epithelium in murine models, thereby offering protective effects against UC.^41^ Additionally, an immunotherapeutic approach was devised by loading a miR-146b mimic onto mannose-modified trimethyl chitosan-conjugated nanoparticles (MTC-miR146b) to selectively target intestinal macrophages for mucosal regeneration and tumor suppression in mouse models, inducing a transition of M1 macrophages to M2 type to inhibit inflammation and promote mucosal regeneration.^68^

Oral nucleic acid drugs are explored as a promising approach for the treatment of UC, for them exhibiting the ability to suppress inflammation and promote healing of the colonic mucosa in UC patients.^69^ Nevertheless, the efficient delivery and distribution of oral nucleic acid drugs to the colon present significant challenges. The administration of miR-200 via the oral delivery of lipid nanoparticles has been shown to effectively restore ISCs and facilitate intestinal regeneration in mice with acute injury. This approach holds promise for the maintenance of intestinal epithelial homeostasis and promotion of regeneration in patients with active UC.^70^ A kind of multistage-responsive nanocomplexes (MSNs) based on polymeric nanocapsules and alginate that used miR-320 as a model nucleic acid drug is synthesized by Li et al. The oral administration of these MSNs effectively releases miR-320 nanocapsules in the colonic cavity, subsequently inhibiting the phosphorylation of IκBα and AKT, which thus reduces colon inflammation, enhances mucosal repair capability, and successfully alleviates UC symptoms.^71^

At present, some anti-UC drugs targeting ncRNAs have been used in clinical studies. Obefazimod, a small molecule that selectively upregulates miR-124 in immune cells, has finished a phase 2b clinic trial for moderate-to-severe, active UC. The detection results revealed that obefazimod significantly improved moderate-to-severe, active UC compared with a placebo, and a phase 3 clinical program is ongoing.^72^ Mastiha, a natural dietary supplement, demonstrated significant efficacy in immune-mediated UC. Following two double-blind, placebo-controlled randomized clinical trials with mastiha, the treatment of mastiha is proved to effectively prevent the increase of miR-155 levels in patients with recurrent UC.^73^

In conclusion, numerous studies have clearly demonstrated that ncRNAs are abnormally expressed in UC and play a crucial role in regulating the intestinal barrier, immune response, and host–microbe interactions. They are stably present in body fluids and tissues, highlighting their potential as noninvasive diagnostic and monitoring biomarkers. However, most of the existing evidence focused on the correlation rather than the causality of the biomarkers and their effects on UC. Moreover, the lack of functional validation through cell-specific knockout or overexpression models make the challenges in the clinical translation of this field. It is also difficult to distinguish the core regulators from the large number of different ncRNA groups. Although the ceRNA mechanism is theoretically intriguing, strict validation in the complex RNA network through CRISPR and other gene editing techniques is still required. Additionally, some results are contradictory such as the dual role of miR-31 in inflammation, which may be due to cell type-specific effects or model backgrounds.^23,34^

Future research should focus on examining ncRNA characteristics in larger-scale and broader populations to verify these findings and perhaps discover more ncRNAs that are helpful for diagnosis. Additionally, a targeted technology system for precisely delivering ncRNAs to specific intestinal cell types has not yet been established. Overcoming the two major bottlenecks of mechanism verification and targeted delivery is a key prerequisite for realizing clinical translation application.

### DNA Methylation in UC

#### DNA Methylation Profiles

DNA methylation represents a prevalent and highly dynamic epigenetic modification that occurs without altering the base sequence.^74^ In eukaryotic organisms, the predominant form of DNA methylation involves the addition of a methyl group to the fifth carbon of the cytosine ring, catalyzed by the family of DNA methyltransferase enzymes (DNMTs), resulting in the formation of 5-methylcytosine (5mC).^75^ In normal cells, DNA methylation modifications predominantly occur at cytosine phosphate guanine (CpG) islands, which are commonly located in regulatory regions, such as gene promoters or enhancers. Although there are tissue-specific variations, approximately 70–90% of dispersed CpG sites exhibit hypermethylation; the majority of CpG islands (CGIs) near promoter regions maintain developmental hypomethylation patterns.^76^ Three stages are involved: DNA methylation, establishment, maintenance, and demethylation. The establishment of DNA methylation is also called de novo DNA methylation, which is responsible for DNMT3. DNMT3 is divided into DNMT3A, DNMT3B, and DNMT3L, among which DNMT3A and DNMT3B are reported to have methyltransferase activity directly. The role of DNMT3L in DNA methylation is maintaining DNMT3A stability.^77^ Unlike de novo DNA methylation, symmetric CpG methylation must be maintained through DNMT1-mediated DNA replication. In the process of it, when the methyl group on a single strand of DNA is lost, DNMT1 plays a role in recognizing the semimethylated DNA and adding the same methylation modification as the template strand.^78^ Ubiquitin protein ligase UHRF1, a multidomain protein E3, can maintain DNA methylation levels by binding to the semimethylated CpG site.^79,80^ DNA demethylation occurs when DNA-binding domains are fused with DNA demethylases, such as ten-11 translocation (Tet) enzymes and thymine DNA glycosylase, which successfully leads to the enhancement to transcriptional responsiveness at targeted loci.^81^

#### Hypermethylation and Hypomethylation Coexist in UC

The correlation of DNA methylation to UC pathogenesis has been well-established.^82^ Although various epigenetic mechanisms may contribute to the development, progression, and maintenance of UC, DNA methylation is the only mechanism consistently shown to be inherited through multiple cell divisions, thereby enabling the permanent transmission of epigenetic information throughout an individual’s lifetime.^83^ Aberrant DNA methylation is frequently observed in the process of UC detected by DNA methylation sequencing and microarrays (Table 3). Hypomethylation has been identified as a factor in the pathogenesis of UC, as described by Gloria et al. in 1996.^84^ Subsequent researchers found several hypomethylated genes associated with inflammation in UC patients.^85^ Of course, it does not mean that no DNA hypermethylation occurred in UC. Based on genome-wide DNA methylation scanning, hypermethylated genes were observed to be involved in homeostasis and defense mechanisms, whereas hypomethylated genes were implicated in immune response.^86^ Moreover, the data from epithelial cells, part of the identified hypo-methylation genes were proved as tumor suppressors in colorectal adenocarcinoma.^87^ The aberrant methylation of the promoter CGIs is strongly correlated with an increased risk of developing colon cancer.^88^ Several aberrant methylated genes have the potential to be biomarkers of UC-associated cancer. Age-related modifications in DNA methylation may account for the phenotypic differences observed in IBD between childhood and adult onset. Furthermore, the observed correlation between CD8+ T cell gene transcription, DNA methylation, and age within the patient cohort indicates that this mechanism may potentially contribute to the age-related variations in IBD phenotype.^89^ Together, DNA methylation constitutes an alternative regulatory mechanism influencing gene expression in the pathogenesis of UC.

**Table 3:** Clinical Value of DNA Methylation in UC (Study Type: Clinical)^a^

years	numbers of clinical samples	sample sources	DNA methylation	clinical value	ref.
2021	control (*n* = 8)	mucosal biopsies	hypo: IL10, SIGLEC5, CD86, et al.	useful biomarkers and targeted treatment strategies for severe UC patients	^85^
	treatment-naïve severe UC (*n* = 8)				
	treatment-naïve mild UC (*n* = 8)				
2017	cancer-free UC (*n* = 84)	mucosae of the rectum	hyper: SOX17, CDH13, DPYS, et al.	correlated with the disease duration and distinguishes severe phenotype UC	^88^
	UC with neoplastic lesions (*n* = 12)				
2012	control (*n* = 11)	colonic mucosa	hypo: RASGRP1, CDC42BPB, PRKCB, et al.; hyper: RNOS, CCND1, COL4A2, etc.	exploited as UC-associated carcinogenesis risk predictors	^90^
	UCM (*n* = 14)				
2014	control (*n* = 10)	rectal mucosa	hyper: CDX1, miR-1247, CDH1, etc.	the methylation status of EMT-related genes is associated with more severe clinical phenotypes in UC	^91^
	UC (*n* = 86)				
2017	UC (*n* = 86)	colonic mucosa	hyper: MINT2 and 31, P16, NEUROG1, etc.	build the link between Fusobacterium enrichment and DNA methylation accumulation in the inflammatory colonic mucosa in UC	^92^
2014	control (*n* = 22)	colonic biopsies	hyper: IFITM1, ITGB2, S100A9, etc.	promote the diagnostic and therapeutic modalities for pediatric UC based on DNA methylation	^93^
	UC (*n* = 9)				
	CD (*n* = 15)				
2023	control (*n* = 85)	rectal mucosa	hyper: PARP1, LIMK1, IFT81, et al.; hypo: GXYLT2, AUTS2, FOXN3, etc.	cell type-specific epigenetic changes are associated with UC severity and outcome	^94^
	UC at diagnosis (*n* = 211)				
	UC at follow-up (*n* = 73)				
2022	UC (*n* = 64)	whole blood of children	RUNX3	methylation of RUNX3 promoter 2 in whole blood DNA is not associated with the characteristics of UC in children	^95^
2020	control (*n* = 3)	colon tissues	S100A9	a potentially predictive biomarker in UC	^96^
	UC (*n* = 3)				
2021	control (*n* = 40)	tissue specimens from patients	hypo: COX-2; hyper: MINT1	the hypomethylation of COX-2 gene is a risk factor through which UC patients are susceptible to colorectal cancer, and the hypermethylation of MINT1 is a risk factor for colorectal cancer but not for UC	^97^
	SUC (*n* = 50)				
	LUC (*n* = 52)				
	SCRC (*n* = 58)				
2020	UC (*n* = 159)	rectum inflammatory mucosa	hyper: CDKN2A	link MIF genotypes and carcinogenesis promotion in UC	^98^
2019	control (*n* = 30)	inflammatory mucosa	RUNX3 and COX2	the methylation rates of RUNX3 and COX2 were not significantly different compared with healthy subjects in the early stage of UC	^99^
	UC (*n* = 41)				
	CRC (*n* = 32)				
2020	control (*n* = 43)	peripheral blood	TGFβ1	a high discriminative power for identifying UC and serves as a diagnostic marker	^100^
	UC (*n* = 31)				
	CD (*n* = 67)				
2014	control (*n* = 12)	biopsy samples	hyper: miR-124a-3	a promising marker for estimating individual risk for UC-associated cancer	^101^
	UC without CAC (*n* = 40)				
	UC accompanied by CAC (*n* = 4)				
	S-CRC (*n* = 8)				
2018	UC (*n* = 4)	colonic biopsy samples	hyper: TUBB6	a potential biomarker for UC-associated dysplasia	^102^
2016	control (*n* = 3)	biopsy samples	hyper: FAM217B, KIAA1614, and RIBC2	new clinical information for the diagnosis and therapeutic treatment of UC	^103^
	UC (*n* = 8)				
2020	UC-CACRC (*n* = 7)	mucosa samples	hyper: CRHR2	promising for cancer screening in UC patients	^104^
2014	control (*n* = 30)	biopsies	hyper: FOXE1 and SYNE1	a useful marker of neoplasia in long-standing UC	^105^
	IBD (*n* = 93)				

a Annotation: UCM: non-neoplastic UC, SUC: the short disease course UC, LUC: the long disease course UC, SCRC: sporadic colorectal cancer, S-CRC: sporadic colorectal cancer, CACRC: colitis associated colorectal cancer.

#### Mechanisms of DNA Methylation in UC

DNA methylation plays a critical role in the integration process and influences multiple facets of UC. The reported mechanisms refer to immunoreaction, cell proliferation, intestinal flora imbalance, and transformation of UC into cancer.

##### Immunoreaction

The demethylation of DNA is revealed to upregulate the expression of EBI3, which results in the formation of anti-inflammatory IL-35.^106^ Tumor necrosis factor superfamily member 13 (TNFSF13) has been recognized as a pivotal regulator in the development and differentiation of B cells. The silencing of DNMT3a in IECs results in decreased methylation levels of TNFSF13, thereby promoting the differentiation of anti-inflammatory CD1d+ B cells in vitro. This process concurrently suppresses proinflammatory responses, thereby mitigating inflammation.^107^ Another research claimed that the dysregulated expression of IL-17 is regulated by UBC9 promoter hypermethylation.^108^ Xu et al. reported DNA hypomethylation of ZBTB7B promotes the production of inflammatory cytokines and exacerbates colonic inflammation in UC, by activating the maturation of CD4^+^ T cells and repressing the differentiation of CD4^+^CD8^+^ T cells.^109^ A comprehensive genome-wide DNA methylation analysis was conducted on CD4^+^ T cells associated with IBD. The methylation patterns of these CD4^+^ T cells effectively differentiated patients with Crohn’s disease from healthy controls. In comparison to regulatory Tregs, the majority of Th17-related genomic regions in Th17 cells, characterized by open chromatin, exhibited hypomethylation and engaged in enhancer–promoter interactions.^110^ DUSP2, which is downregulated by DNA methylation in clinical UC patients, was proved to act as STAT3 phosphatase, thereby modulating the development of TH17 cells in immune response and inflammation.^111^ Interestingly, ncRNAs are discovered in the process of DNA methylation, thereby modulating the development of TH17 cells in immune response and inflammation. Several inflammatory immune response genes are found downstream of DNA methylation regulating lncRNAs, including SERPINB1, CCL18, and SLC15A4.^112^

##### Cell Proliferation

The abnormal DNA methylation is linked to the inhibition of epithelial cell proliferation and damage to the intestinal barrier during UC. The inhibition of DNMT3A-mediated SMARCA5 methylation is reported to inactivate Wnt/β-catenin signaling pathway-mediated cell proliferation and apoptosis, which leads to intestinal barrier dysfunction and finally aggravates UC.^113^ Another study demonstrated that increased methylation of the CBS promoter enhances epithelial cell injury induced by TNF/IFN through the activation of NF-κB p65-mediated MLCK-P-MLC signaling.^114^

##### Intestinal Flora Imbalance

Furthermore, a combined RNA-seq and Whole Genome Bisulfite Sequencing (WGBS) analysis of colonic crypt epithelial cells revealed that the microbiota causes the changes in DNA methylation, thereby leading to alterations in the expression programs of genes related to colitis.^115^ In the UC models, it has been revealed that regulating the gut microbiota could alter the status of DNA methylation of the host.^116^ The accumulating evidence indicates that gut microbiota modulate metabolite availability, thereby influencing DNA methylation patterns.^117^ For instance, microbial-derived propionic acid was reported to reduce the production of IL-6 in monocytes by attenuating the methylation in CpG-rich intronic regions of IL-6 gene, thereby mitigating inflammatory responses.^118^ Although the precise mechanisms by which gut microbiota influence DNA methylation remain to be fully elucidated, the role of the bidirectional host–microbe interaction axis in UC is an intriguing aspect.

##### The Transformation of UC into Cancer

The conversion to cancer during UC development has also been reported to involve DNA methylation. The suppression of ELF4 by DNA hypomethylation in UC is proved to predispose the host to colorectal cancer.^119^ Ibrahim et al. revealed a pathway that DNMT3b-dependent DNA hypermethylation at the IRF8 promoter, which is purposed by IL-10, builds a link between UC and colon cancer initiation.^120^ Moreover, the methylation of the BVES promoter is reported to be associated with the promotion of UC-derived tumorigenesis through the dysregulation of Wnt and c-Myc signaling.^121^ The aforementioned studies elucidated the mechanisms through which DNA methylation affects the UC.

#### Medications Used to Treat UC Affect DNA Methylation

Only a few drugs have been reported in the literature to treat UC by regulating DNA methylation. An in vivo experiment revealed that exosomes derived from cow and human milk can ameliorate UC targeting the downregulation of DNMT1 and DNMT3.^122^ In addition, an antibiotic cocktail (ciprofloxacin, metronidazole, neomycin, and vancomycin) was reported to suppress UC-associated tumorigenesis inhibiting inflammation-induced aberrant DNA methylation.^116^ Alpinetin, a flavonoid compound, has been demonstrated to restrain the expression of DNMT1 and the methylation level of Foxp3 promoter region, thereby promoting the differentiation of regulatory Tregs and then exerting anti-colitis effects.^123^ However, the clinical value of these studies is insufficient, and the development of anti-UC drugs targeting DNA methylation remains challenging.

DNA methylation plays a certain role in UC by modulating immune responses, epithelial barrier integrity, host–microbiome interactions, and carcinogenesis. The abnormal DNA methylation contributes to disease chronicity and the formation of preneoplastic fields.^124^ However, unlike genetically tractable ncRNAs, the global inhibition of DNA methyltransferases, such as 5-azacytidine, would induce genome-wide hypomethylation and complicate phenotypic interpretation.^125^ The lack of efficient and specific “DNA methylation surgery” tools continues to impede causal studies and therapeutic targeting of DNA methylation in UC.

### Histone Modification in UC

#### Histone Modification and Its Transcriptional Regulatory Function

Histones, consisting of globular and tail domains, are a kind of octamer comprising an H2A-H2B tetramer and two H3–H4 dimers.^126^ Histones tightly coil 146–147 DNA base pairs, which makes the post-translational modifications of the N-terminal and C-terminal tails of histones alter histone–DNA interactions, resulting in either transcriptional activation or gene silencing.^127^ These modifications, especially methylation and acetylation, play a crucial role in modulating the chromatin structure and function, thereby influencing the transcriptional activity of genes.^128^ The significance and mechanisms of histone modifications in UC are gradually being revealed.

Acetylation and methylation represent the most extensively investigated forms of histone modification in the context of UC.^129^ Histone acetylation entails the enzymatic addition of an acetyl group (COCH_3_) to positively charged lysine residues by histone acetyltransferase (HAT), resulting in tight binding to the negatively charged phosphate group. This process reduces the electrostatic interactions between the histones and the negatively charged phosphate backbone of DNA, thereby reducing chromatin compaction and promoting a more relaxed chromatin structure conducive to gene transcription.^130^ Conversely, the removal of the acetyl group regulated by histone deacetyltransferase (HDAC) promotes chromosome compression and inhibits transcription.^128^ Slightly differently, histone methylation primarily targets lysine residues on histones H3 and H4 by adding methyl groups, predominantly affecting the side chains of lysine and arginine residues.^131^ The effects of histone methylation depend on the specific location and state of the modification. Placing methylation at different locations within the tail structure domain of histones can alter the chromatin structure by either enhancing or reducing DNA–histone interactions, thereby impacting gene transcription activation or repression.^132^ Histone methyltransferase (HMT) catalyzes methylations using S-adenosyl methionine (SAM) to transfer methyl groups to histone lysine residues.^133^ Histone methylation is traditionally believed to be a permanent modification; however, recent findings indicate that histone demethylation is a complex and tightly regulated process. Demethylases can be classified into two main families based on their substrates and mechanisms of action: lysine-specific demethylase and Jumonji domain-containing protein D3 (JMJD3) demethylase. The intricate equilibrium between histone methylation and demethylation plays a crucial role in determining the transcriptional activity of genes.^134^

#### Mechanisms of Histone Modification in UC

Histone modification is recognized for its ability to modulate gene transcription and contribute to the development of abnormal immune responses in the pathogenesis of UC. As shown in Table 4, similar to other epigenetics, the differences in histone modification could be used as the biomarkers of inflammatory cancer transformation, disease diagnosis, and disease prognosis. In individuals afflicted with UC, the expression of HDAC1 and HDAC7 serves as critical indicators for both diagnosis and prognosis in the pathogenesis of colorectal cancer.^135^ The studies of histone modification not only involved in the expression of modified enzymes, the literature has reported the changes of histone modification profile. A total of 253 histone modification-related differentially expressed genes were identified between inflammatory and noninflammatory patients with UC. Among these, the downregulation of CAMK2D was observed in individuals with inflammatory UC, which has also been identified as a significant predictive biomarker for response to infliximab therapy.^136^ The different levels of H3K27me3 modification from peripheral blood samples are proved to be a diagnostic marker to distinguish UC and CD.^137^ The level of histone H3 acetylation is markedly reduced in the colonic epithelium of individuals with UC and exhibits a negative correlation with the severity of the disease.^138^ Histone H3K9 acetylation and histone H3K18 lactylation are revealed to be associated with the downregulation of macrophage pyroptosis, which is regarded as a treatment strategy for UC.^139^ In addition, a study has demonstrated that PAD4 facilitates histone citrullination, leading to the formation of mucosal ulcers in neutrophil extracellular traps and subsequent conversion of blood clots into immunothrombosis, ultimately mitigating the severity of mucosal bleeding and ulcers.^140^ Within IECs, the citrullination of mitochondrial creatine kinase 1 (CKMT1) at the R242 site by PAD4 results in a reduced stability of the CKMT1 protein via the autophagy pathway. This process disrupts mitochondrial homeostasis, compromises intestinal barrier integrity, and triggers apoptosis in IECs.^141^

**Table 4:** Clinical Value of Histone Modifications in UC (Study Type: Clinical)^a^

years	numbers of clinical samples	sample sources	histone modifications	clinical value	ref.
2023	control (*n* = 11)	peripheral blood		the four input features of epigenetic subsets originating from immune cell types (HPC, NK1, and GD3) demonstrate the ability to effectively differentiate between patients with IBD and healthy individuals, irrespective of the specific subtype or severity of colitis	^16^
	IBD (*n* = 83)				
2015	control (*n* = 20)	tissue samples	histone modification enzyme	NEK6, AURKA, HDAC1, and PAK1 exhibited significant upregulation in individuals with an extended history of UC. Therefore, HDAC1, PAK1, NEK6, and AURKA may serve as potential diagnostic indicators for colorectal cancer screening in patients with UC	^135^
	UC (*n* = 20)				
	CRC (*n* = 20)				
2023	UC-noninflammation (*n* = 20)	colon biopsy		histological severity of colon biopsy negatively correlated with CAMK2D protein expression levels	^136^
	UC-inflammation (*n* = 20)				
2019	control (*n* = 50)	peripheral blood samples	H3K27me3 modification	FBXW7 expression is markedly increased in inflamed intestinal tissues from patients with UC or CD and may serve as a new diagnostic marker	^137^
	IBD (*n* = 68)				
2022	UC (*n* = 36)	biopsy samples	histone citrullination	facilitate the recruitment of neutrophils to the fibrin layer, leading to the initiation of secondary immunothrombosis and ultimately serving as a protective mechanism against colonic bleeding in individuals with active UC	^140^
2021	control (*n* = 47)	colonic mucosa	histone H3 acetylation	significantly lower in UC colon epithelium and negatively associated with disease severity	^142^
	active UC (*n* = 45)				
	nonactive UC (*n* = 32)				
2018	control (*n* = 75)	stool samples		reduce genetic capacity of colonic microbiota to produce butyrate	^143^
	UC (*n* = 58)				
	CD (*n* = 71)				
2019	control (*n* = 10)	biopsies samples	HDAC	ANP32E enhances glucocorticoid receptor-mediated transcription by interacting with the histone variant H2	^144^
	UC (*n* = 24)				
2017	UCHR (*n* = 18)	biopsies samples		frequent mutations in the ARID1A (44%), SMARCA4 (17%), MLL2 (44%), MLL3 (67%), SETD2 (17%), and TET2 (50%) genes associated with histone modification and chromatin remodeling were identified in individuals with undifferentiated carcinoma with clear cell features	^145^
2016	control (*n* = 25)	biopsies samples		the expression of lysine acetyltransferase 2B (KAT2B) was found to be significantly reduced in colon tissues from patients with inflammatory UC compared to noninflammatory tissues from healthy controls and patients with IBD	^146^
	UC (*n* = 24)				
	inflammatory CD (*n* = 22)				
	noninflammatory CD (*n* = 26)				

a Annotation: UCHR: long-standing UC subjects at high risk of colorectal carcinoma.

#### The Interaction between Gut Microbiota and Histone Modification in UC Pathogenesis

Commensal bacteria could metabolizes dietary fibers to produce various metabolites, such as butyrate and propionate, function as substrates or regulators of epigenetic enzymes.^147^ Butyrate acts as a histone deacetylase inhibitor (HDACi) and could affect intestinal immune cells and enhance chromatin accessibility.^148^ It has also been reported to promote the differentiation and function of regulatory T cells (Tregs) by facilitating Foxp3 expression, contributing to immune homeostasis.^149,150^ In IECs, butyrate could inhibit HDAC8-mediated deacetylation of NF-κB p65, upregulating Slc26a3 and tight junction proteins to reinforce mucosal barrier integrity—a mechanism particularly relevant in (UC).^151^ Conversely, colitis-associated microbiota dysbiosis leads to the chaos of microbial metabolites and decreased histone acetylation, resulting in the silencing of protective genes of UC by decreasing histone acetylation in cells. This establishes a vicious cycle wherein inflammation alters microbiota composition and exacerbates epigenetic dysregulation.^152^ Other microbial metabolites, including secondary bile acids such as tauroursodeoxycholic acid, deoxycholic acid, and lithocholic acid, are also implicated in HDAC regulation, though their specific roles in UC remain to be fully elucidated.^153,154^ Collectively, the microbiota metabolite could modulate histone modification, thus involving the pathogenesis of UC.

#### Medications Used to Treat UC Affect Histone Modification

Enhancer of zeste homologue 2 (EZH2), a member of the methyltransferase family, is considered a pivotal regulator of chromatin condensation through the canonical trimethylation of H3K27me3, facilitating the expression of autophagy-related protein 5, followed by the degradation of NLRP3, thus inhibiting UC.^155^ Moreover, the reduction of EZH2 ubiquitination results in an increase in H3K27me3 modification, leading to a suppression of the recruitment of proinflammatory macrophages to colonic inflammatory tissues, thereby conferring protection against inflammation and disease progression in mice with colitis.^137^ JMJD3, a demethylase targeting H3K27me3, has been implicated in ameliorating immune dysfunction within the intestinal mucosa. Inhibition of JMJD3 has been demonstrated to suppress the secretion of inflammatory mediators and modulate the differentiation of Th17 and Treg cells, thereby alleviating inflammation in a murine model of acute UC induced by dextran sulfate sodium DSS.^156^

HDAC2 functions as a negative regulator of the Wnt signaling pathway by binding to the promoter region, thereby promoting goblet cell differentiation.^157^ HDAC3 in the intestinal epithelium is sensitive to microbial flora and could regulate the homeostasis of IECs and immune cells. Butyrate regulates intestinal type 2 immunity by restricting the differentiation of cluster cells, a secretory type of intestinal epithelial cells, through histone deacetylase 3.^158^

HDACis are widely reported for their regulatory role and mechanisms in UC. The administration of givinostat and vorinostat, the members of HDACis, is demonstrated to improve the transepithelial resistance and then inhibit the macromolecules across epithelial monolayers under inflammatory conditions. In mice with DSS-induced chronic colitis, the administration of HDACi has been shown to modulate immunity and improve colitis pathology.^159^ The inhibition of HDAC could promotes tissue repair by enhancing epithelial cell recovery and accelerating migration. An in vivo study showed that the treatment of givinostat enhanced mucosal repair and intestinal regeneration following barrier disruption and mechanical damage induced by DSS.^160^ Furthermore, HDACis have been shown to significantly increase the expression of EBI3 in human colonic epithelial cells, which is a regulator that promotes the production of anti-inflammatory factors. WT161, an inhibitor of HDAC6, is confirmed to impede the activation of NLRP3 inflammasome by disrupting ASC spot formation and decreasing NLRP3 expression, mitigates intestinal damage, and suppresses intestinal inflammation in colitis models. These effects of WT161 are revealed to play roles in mitigating intestinal damage and suppressing intestinal inflammation in active UC.^161^ Similarly, C646, a histone acetyltransferase p300 inhibitor, has been shown to impede the assembly of the NLRP3 inflammasome by disrupting the interaction between NLRP3 and ASC, thereby exhibiting anti-inflammatory properties in DSS-induced UC.^162^ An ongoing study has shown that the treatment with MS-275, a specific HDAC1/3 inhibitor, inhibits histone H3 deacetylation, thereby attenuating NF-κB-induced inflammation, migrating apoptosis, and preserving epithelial barrier function, thereby reducing UC.^138^ The long-term use of low-dose ASA inhibits protein expression and activity of HDACs through epigenetic mechanisms, resulting in a significant increase in the enrichment level of H3K37ac in the gene promoter region. This serves as a foundational mechanism by which ASA prevents UC.^163^

However, the current clinical studies of HDACis for the treatment of UC have not gone well. A randomized, open-label, two-arm, multicenter phase-II study will be conducted to investigate the potential impact of incorporating an orally active HDACi-valproic acid into first-line bevacizumab/oxaliplatin/fluoropyrimidine regimens on progression-free survival in patients with RAS-mutated metastatic colorectal cancer.^164^

Another important aspect of future research is that histone modifications act as a dynamic bridge in the interaction between environmental factors, especially in microbial metabolites, and the transcriptional programs that regulate intestinal immune and barrier functions. The therapeutic effects of HDACis in most preclinical colonic inflammation models highlight the significance of this regulatory layer. However, due to the technical complexity of chromatin analysis, techniques like ChIP-seq require numerous cell samples, which makes them difficult to apply to precious clinical biopsy samples and unable to distinguish specific effects of cell types in inflamed tissue environments. Furthermore, although current research emphasizes histone-modifying enzymes (writers and erasers), the contributions of effector proteins that interpret these marks remain poorly characterized. Moreover, the functional outcomes of histone modifications are highly context-dependent, exemplified by the cell-specific roles of HDAC3, resulting in pleiotropic and often contradictory effects that complicate targeted therapeutic intervention.^165^

#### RNA Modifications

RNA modification alters the chemical composition and molecular structure of RNA through the addition of various chemical groups, such as N6-methyladenine (m6A), N1-methyladenosine (m1A), and 5-hydroxymethylcytosine (hm5C).^166^ Among these modifications, m6A is the most prevalent and widely studied, particularly in relation to its association with the pathogenesis of UC.^167^ The m6A modification of RNA is facilitated by a methyltransferase complex composed of methyltransferase 3 (METTL3), methyltransferase 14 (METTL14), and Wilms tumor 1 binding protein and can be reversed by the demethylases fat mass associated and obesity associated protein (FTO) and alkB homology 5.

#### The Mechanism of RNA Modification in UC

It has been demonstrated that the absence of METTL14 in IECs compromises the integrity of the cell barrier, by hindering the maintenance of self-renewal in colon epithelial homeostasis. Mechanistically, inhibitory κB (IκB) protein Nfkbia serves as the direct target of m6A modification by METTL14 in colonic epithelial cells.^168^ Moreover, the deficiency of METTL14 in T cells is reported to propel the dysregulation of Treg cells and then promote spontaneous colitis in mice.^169^ M6A irCLIP-Seq analysis was conducted on CD4+ T cells derived from METTL14^–/–^ mice, demonstrating a reduction in the level of m6A modification within select *Socs* gene transcripts. This reduction resulted in heightened *Socs* mRNA stability, elevated levels of SOCS proteins, and inhibition of the IL-2-STAT5 signaling pathway, finally exaggerating immune response characteristic of intestinal inflammation.^170^ Coptisine, a natural small molecule substance derived from *Coptis chinensis*, exhibits anti-UC properties by upregulating METTL14 expression via improving the stability of TSC1 mRNA, suppressing M1 macrophage polarization.^171^

A study demonstrated a reduction in the expression levels of the M6A-binding protein IGF2BP2 in tissue samples from patients with UC in comparison with those from healthy individuals. Moreover, it has been observed that the upregulation of m6A phenotype-related hub genes, specifically NUP37, SNRPG, and H2AFZ, correlates with elevated levels of M1 macrophages, M0 macrophages, and naive B cells in individuals with IBD.^172^ Another research indicates the ability of IGF2BP2 to enhance the stability of GPX4 mRNA through m6A modification, leading to a reduction in ROS expression, inhibition of ferroptosis, and exertion of an anti-UC effect.^173^ Another demethylase, FTO, has been implicated in the maintenance of intestinal homeostasis, leading to an increase in m6A modification and a concomitant decrease in the mRNA stability of CerS6, which encodes ceramide synthetase. Subsequentially, the release of ceramide by IECs can trigger proinflammatory macrophages to secrete serum amyloid A protein 1/3, thus promoting the differentiation of Th17 cells and exacerbating colitis.^174^

Recently, N7-methylguanosine (m7G) has been identified as a novel RNA epigenetic modification with potential implications for prognostic prediction in intestinal malignant tumors and evaluation of the tumor immune microenvironment.^175^ Five m7G-related genes (NUDT7, NUDT12, POLR2H, QKI, and PRKCB) were identified to show the diagnostic potential for UC. Moreover, gene modules that exhibit a strong correlation with the expression of m7G hub genes were found to be enriched in pathways related to inflammation.^176^

RNA modifications, particularly m6A, serve as crucial post-transcriptional regulators in UC, modulating mRNA stability and translation to influence epithelial integrity and immune responses. However, this field still faces several limitations. The current transcriptome-wide mapping techniques such as MeRIP-seq lack single-base resolution and fail to reliably detect modification sites on low-abundance transcripts.^177^ Moreover, the functional relevance of most identified m6A sites remains unvalidated, and research emphasis remains disproportionately focused on m6A, overlooking other widespread modifications, including m7G, m1A, and uridylation, that may play significant roles in UC.

#### Multilayer Epigenetic Coordination in UC

Multiple epigenetic mechanisms jointly influence the function of key genes or signaling pathways through hierarchical regulation, thereby participating in the occurrence and development of UC. From the current research perspective, ncRNAs seem to be the core regulatory hub in this process. ncRNAs could integrate and coordinate multiple layers of epigenetic information, forming a complex cross-dialogue network.^178^ The studies have shown that circ-CCND1 could bind to histone demethylase (KDM6B), increasing the level of H3K27me3 in the promoter region of ELF3. Thus, the expression level of ELF3 is downregulated, and thereby promoting the expression of miR-342-3p, which then specifically inhibits KDM6B, ultimately alleviating DSS-induced intestinal epithelial cell pyroptosis.^179^ Meanwhile, miR-182-5p could target DNMT3A, reduce the methylation level of SMARCA5 gene, and inhibiting the Wnt/β-catenin signaling pathway, thereby exacerbating the progression of UC.^113^ The hypermethylation of differentially methylated regions is often accompanied by the downregulation of adjacent lncRNA expression, but the reverse regulatory mechanism remains to be further explored.^112^

Also, other types of epigenetic regulation exhibit interactions with each other. There is a close and synergistic relationship between DNA methylation and histone modification.^180^ For instance, DNMT3B could be recruited to specific chromatin regions by histone H3 modifications to perform the function of DNA methylation. The abnormal assembly of this complex such as DNMT3B mutation or changes in histone modification status could lead to dysregulation of DNA methylation and promote the occurrence of UC.^181^ Different epigenetic mechanisms could also work synergistically to influence the expression levels of the downstream genes or proteins. METTL3-mediated m6A modification is reported to increase the stability of Kcnk6. Interestingly, the process of histone lactylation also activates the transcription of Kcnk6. This coordinated effect of multiple epigenetic mechanisms has facilitated the process of inflammation-associated carcinogenesis.^182^ RNA modifications are also involved in this regulatory network. METTL14, as a core component of the m6A methyltransferase, could mediate the stability of lncRNA DHRS4-AS1 and then alleviate colonic inflammation through the DHRS4-AS1/miR-206/A3AR axis.^183^

In conclusion, epigenetic regulation in UC is a dynamic and interconnected network system (Figure 3). ncRNAs, DNA methylation, histone modifications, and RNA modifications collectively regulate the inflammatory response, epithelial barrier function, and immune homeostasis through cooperative or antagonistic effects. Currently, research studies have not achieved a precise and comprehensive analysis of this network. Further elucidation of the molecular regulatory networks of different epigenetic mechanisms would provide new intervention strategies for the treatment of ulcerative colitis.

**Figure 3:**
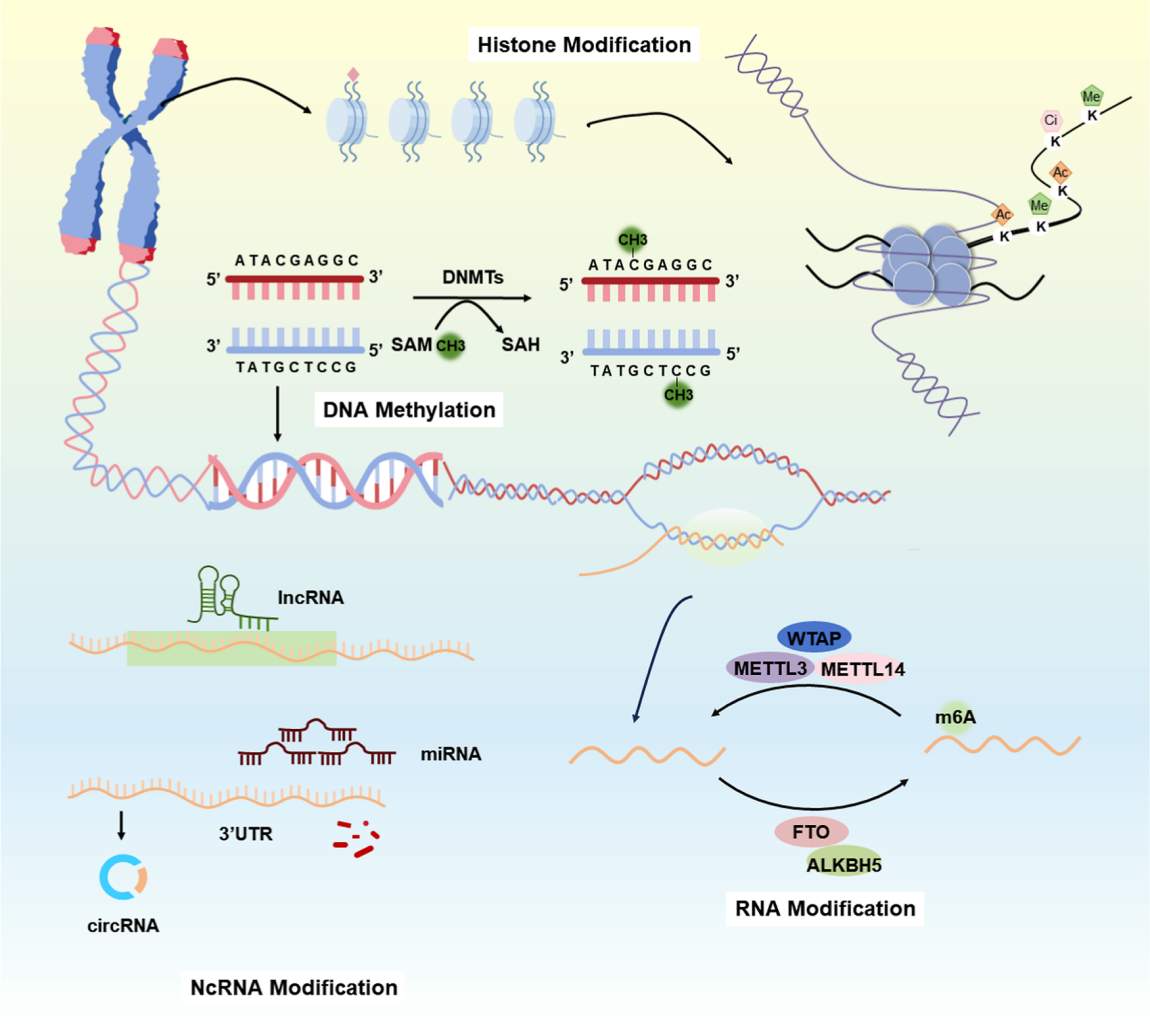
Epigenetic mechanisms. Histone modification, DNA methylation, ncRNA modification, and RNA modification pathways.

#### Challenges and Future Direction of Epigenetics in UC

Epigenetics research has introduced a novel perspective on the pathogenesis of UC, revealing complex regulatory mechanisms that extend beyond the genetic sequence. However, the clinical translation of these findings encounters several obstacles. ncRNAs, DNA methylation, histone modification, and RNA modification all face the fundamental challenge of establishing the causal relationship of epigenetic changes rather than merely discovering correlations. Furthermore, it is crucial to elucidate their specific roles within distinct cell types, including epithelial and immune cells, because the same epigenetic modification may have completely different effects on UC in different environments. The high degree of cellular heterogeneity complicates the interpretation of results derived from bulk tissue analyses, making identification of driving events difficult. Additionally, part of the application of treatment strategies such as ncRNAs drugs or HDACis is limited by their efficiencies. The off-target effects of these strategies exist from bulk tissue analyses. Future research directions may capitalize on single-cell multiomics and epigenomics technologies to precisely analyze epigenetic regulation at the cellular and tissue microenvironment levels.^184^ Additionally, the development of the novel technologies, such as proteolysis-targeting chimeras and nanodelivery, has brought the goal of achieving precise targeted treatment for UC much closer.^185^ The integration of cross-disciplinary integration and technological advancements is expected to significantly enhance the translation of epigenetic research results in the clinical diagnosis and treatment of UC.

## Discussion

Epigenetics has been associated with a multitude of complex human diseases, and research in this domain has undergone substantial expansion in recent years.^186^ The mounting evidence supports the notion that epigenetic changes play a crucial role in the pathogenesis of UC.^187^ The extensive modifications of DNA, RNA, and histones at the epigenetic level are recognized as significant mechanisms underlying UC. As elucidated in this review, the functions and processes of epigenetic alterations in UC are delineated, encompassing noncoding RNAs, DNA methylation, histone modifications, and RNA modifications (Figure 4).^7^ Collectively, these results support the concept that intricate and reciprocal epigenetic regulatory networks exist in UC, highlighting the potential significance of epigenetic markers in the diagnosis and prognosis of UC.

**Figure 4:**
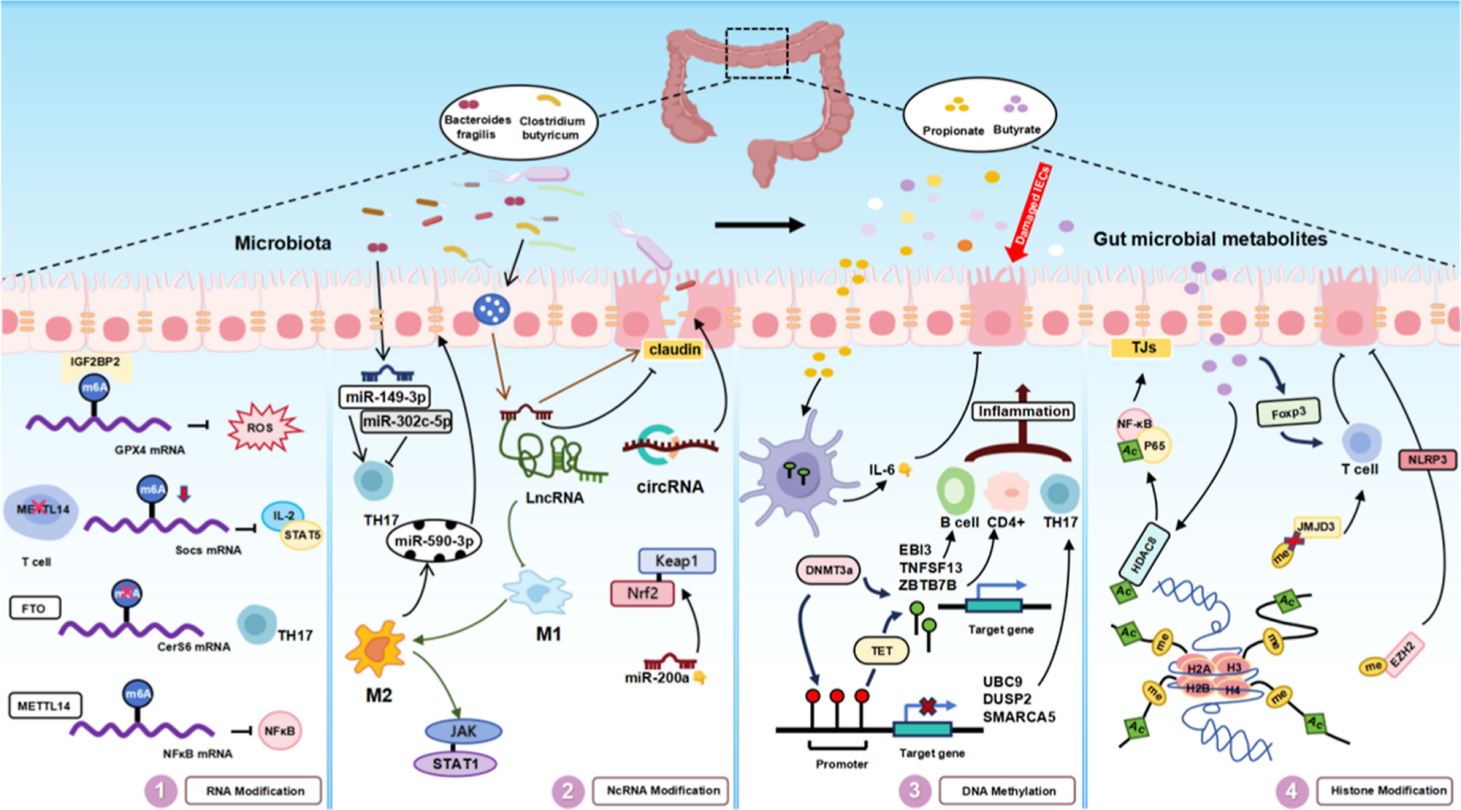
Each epigenetic mechanism interacts with immune cells, IECs, and the gut microbiome in the UC.

Extensive evidence derived from clinical samples, encompassing mucosal tissue and blood, reveals a diverse array of epigenetic alterations in the progression of UC. These alterations have the potential to either facilitate or impede the onset of UC by modulating the immune milieu within tissues and mucosal barriers. Given the propensity of UC to progress to colon cancer, current research indicates that epigenetic assays may serve as a predictive tool for identifying cancer in afflicted individuals. Distinguishing between UC and Crohn’s disease in a clinical setting has long presented a challenge. Certain epigenetic molecules have shown promise as potential markers for differentiation under the two conditions.

The available research indicates that those altered epigenetic molecules may serve as a promising therapeutic target for the treatment of UC (Table 5). Specifically, obefazimod and mastiha, which are designed to target miR-124 and miR-155, respectively, have progressed to clinical trials as potential agents for the treatment of UC.^72,73^ In addition, the efficacy of anti-UC drugs targeting ncRNAs, DNA methyltransferases, and histone deacetylases has been assessed in animal and cellular models. Nevertheless, there is a limited selection of pharmaceuticals designed to target RNA modification in the treatment of UC.

**Table 5:** Epigenetic Targeted Therapeutic Drugs

drugs	epigenetic mechanism	model	targets	efficacy outcome	current stage	major limitations	ref.
limonin	ncRNAs	animals: 3% DSS C57BL/6J male mice.	miR-214	limonin improves the prognosis of UC mainly through downregulating p-STAT3/miR-214 levels and was a novel therapeutic agent and was expected to be translated into the clinic to improve the prognosis of UC	nonclinical	it is worth pursuing whether limonin could regulate other targets through miR-214 as well as whether PTEN and PDLIM2 are also regulated by other miRNAs in the near future	^65^
		cell: NCM460					
cinnamaldehyde	ncRNAs	animals: 5% DSS BALB/c male mice.	miR-21 and miR-155	cinnamaldehyde ameliorates DSS-induced colitis through inhibition of NLRP3 inflammasome activation and miR-21 and miR-155 levels in colons and macrophage, suggesting that CA might be a potentially effective drug for UC	nonclinical	the incompleteness of the mechanism argument, especially the lack of crucial data to establish a direct causal relationship, as well as the failure to rule out the widespread off-target effects that exist within the body	^66^
		cell: RAW264.7 and human monocytes U937					
butyricum-derived extracellular vesicles	ncRNAs	animals: 3% DSS C57BL/6J male mice.	miR-199a-3p	butyricum-derived extracellular vesicles can be a novel agent for the treatment of colitis and miR-199a-3p can be a potential target for IBD treatment	nonclinical	the detailed mechanism through which the gut microbiota affects miRNA expression still needs to be explored because it may be considered a potential therapeutic approach for intestinal-related diseases	^67^
		cell: RAW264.7					
mannose-modified trimethyl chitosan-nanoparticles	ncRNAs	animals: 3% DSS C57BL/6J male mice.	miR146b	MTC-miR146b should be regarded as an effective candidate for oral delivery and could improve the efficacy of immunotherapies for ulcerative colitis and colitis-associated cancer	nonclinical	the oral delivery of miRNAs faces multiple challenges in moving molecules from the gastrointestinal tract to the intracellular space of target cells	^68^
		cell: BMDM					
ABX464	ncRNA	human: moderate-to-severe, active ulcerative colitis	miR-124	all doses of ABX464 significantly improved moderate-to-severe, active ulcerative colitis compared with a placebo, as measured by changes in MMS from baseline to week 8. A phase 3 clinical program is ongoing	phase 2b, double-blind, randomized, placebo-controlled induction trial	one limitation of this study is that we did not test for a dose response effect, and the observed data did not suggest the clear existence of an effect	^72^
mastiha	ncRNA	human: IBD patients	miRNA-155	circulating levels of miR-155, a critical player in the differentiation of Th17 cells, are regulated by mastiha administration in IBD and possibly in NAFLD that share common pathophysiological features, suggesting this as a mediator of mastiha’s anti-inflammatory activities	two double-blinded and placebo-controlled randomized clinical trials	the sample size is relatively small and the differences in microRNA levels among different individuals are significant, which makes it impossible to identify the subtle differences between different groups	^73^
cow and human milk-derived exosomes	DNA methylation	animals: 5% DSS male BALB/c mice.	DNMT1 and DNMT3	human and cow MDEs are up taken by intestine cells, exerting a therapeutic and anti-inflammatory effect in a colitis murine model	nonclinical	the insufficient depth of the mechanism and the significant obstacles to its clinical application	^122^
		cell: PBMCs					
antibiotics	DNA methylation	animals: AOM and 1.5% DSS male BALB/c mice		antibiotics suppressed tumorigenesis through inhibition of aberrant DNA methylation induced by chronic inflammation	nonclinical	the results of such studies may be influenced by various factors, including bacterial strains, bacterial preparations, administration time, and the health condition or status of the mice	^116^
alpinetin	DNA methylation	animals: 2.5% DSS female C57BL/6 mice.	DNMT1	activating AhR, promoting expression of miR-302, downregulating expression of DNMT-1, reducing methylation level of the Foxp3 promoter region, facilitating combination of CREB and the promoter region of Foxp3, and upregulating the expression of Foxp3	nonclinical	insufficient reverse verification of the causal relationship within the body	^123^
		cell: lymphocytes					
GSK-J1	histone modification	animals: 3% DSS male BALB/c mice.	H3K27me3	JMJD3/H3K27me3 epigenetic modification may be involved in the occurrence and development of UC	nonclinical	the exploration of the relevant cellular experimental mechanisms is not deep enough. JMJD3/H3K27me3 may act on certain gene loci within the chromatin	^156^
		cell: Treg cells and Th17 cells					
givinostat and vorinostat	histone modification	animals: 2% DSS mice.	HDAC	a novel tissue regenerative property of the pan-HDAC inhibitors givinostat and vorinostat in intestinal inflammation, which may have beneficial implications by repurposing HDAC inhibitors for therapeutic strategies for inflammatory bowel disease	preclinical	the nonspecificity of HDACi limits the development of precise targeted drugs, as the mechanism is not precise enough. It is impossible to rule out the impact of HDAC inhibition on other related cells	^160^
		cell: human colorectal cell line T84 and murine rectal cell line CMT93					
		human: the colonic mucosa of CD and UC					
WT161	histone modification	animals: 3% DSS C57BL/6J male mice.	HDAC6	in a colitis model, WT161 ameliorated intestinal injury, suggesting its potential use in the treatment of IBD	nonclinical	how WT161 affects the assembly of NLRP3 in the DSS-induced colitis mouse model plays a protective role, and whether WT161 affects the polarization of macrophages in vivo and in vitro remains to be further explored	^161^
		cell: primary peritoneal macrophages					
C646	histone modification	animals: 3% DSS C57BL/6J male mice.	histone acetyltransferase p300	C646 exerts protective effects against colitis induced by DSS by inhibiting the activation of the NLRP3 inflammasome and the NF-κB signaling pathway. C646 may be a candidate drug for the treatment of inflammatory bowel disease	nonclinical	the main limitation of this study lies in the unclear clinical translational potential and the lack of a NLRP3-specific knockout model to directly confirm the effect of the target	^162^
		cell: primary peritoneal macrophages					
aspirin	histone modification	animals: AOM and 1.2% DSS male CF-1 mice	H3K27ac	the preventive effect of aspirin on colitis-associated colon cancer induced by oxaliplatin/sodium dextran sulfate involves the activity of histone deacetylases	nonclinical	using only the CF-1 mouse model, the species differences are limited to those relevant to humans, there is a lack of dose–response relationship and direct mechanism evidence	^163^
valproic acid	histone modification	human: RAS-mutated mCRC patients	HDACi	the “revolution” study aims to improve the treatment efficacy of RAS-mutated mCRC through an attractive strategy evaluating the combination of VPA with standard cancer treatment	a randomized, open-label, two-arm, multicenter phase-II study	the small sample size of the subjects, the nonblinded design may introduce bias, and the lack of a placebo control group limits the reliability of the results	^164^

The complexity of epigenetic regulation in UC is undeniable, as various components such as ncRNAs and DNA methylation interact and influence each other within this network. Certain noncoding RNAs derived from immune cells have been shown to modulate protein function through direct binding, thereby exerting regulatory control over inflammatory signaling pathways and influencing the progression of UC.^47^

Nevertheless, research on epigenetics in UC continues to encounter obstacles, particularly in elucidating the underlying mechanisms of epigenetic alterations in this disease. Current evidence suggests that inflammatory cells exhibit the highest level of epigenetic activity in UC. During the proinflammatory polarization of macrophages, there is an upregulation in the expression of DNA methyltransferase, leading to an overall increase in DNA methylation levels within the cells. Furthermore, the functional alteration of numerous inflammatory cells is concomitant with metabolic reprogramming, whereby altered metabolites can facilitate post-translational modifications of proteins, notably histone acetylation and lactylation, which have been extensively documented in the literature. In addition, the gastrointestinal tract harbors a diverse population of microbes, and numerous studies have documented alterations in the composition and roles of gut microbiota in individuals with UC. One of the pathways through which gut microbiota can impact UC is by releasing vesicles containing ncRNAs that can modulate host cell protein expression.^44,46^

In summary, epigenetic mechanisms play a crucial role in the pathogenesis of UC. Further research into epigenetic modifications is expected to elucidate the more intricate mechanisms underlying the etiology, progression, and treatment of UC, leading to the identification of novel diagnostic markers and therapeutic targets.
